# Disparities in Adverse Perinatal Outcomes Among Pacific Islanders in the Commonwealth of the Northern Mariana Islands

**DOI:** 10.5888/pcd15.170385

**Published:** 2018-03-08

**Authors:** Rica Dela Cruz, Jeanolivia Grant, Julia E. Heck, Haley L. Cash

**Affiliations:** 1 Commonwealth Healthcare Corporation, Saipan, Northern Mariana Islands; 2 Department of Epidemiology, UCLA Fielding School of Public Health, Los Angeles, California; 3 Regional Epidemiology Unit, Pacific Island Health Officers’ Association, Honolulu, Hawaii

## Abstract

**Introduction:**

Although other studies have found evidence for perinatal health disparities among Pacific Islanders in other regions, no studies have evaluated racial/ethnic disparities in adverse perinatal health outcomes in the small US island territory of the Commonwealth of the Northern Mariana Islands (CNMI).

**Methods:**

We used retrospective cohort data on 8,427 singleton births from 2007 to 2014 at the Commonwealth Healthcare Corporation (CHCC), the only hospital in the CNMI. We used multivariate logistic regression to estimate risk for preterm birth (<37 weeks) and macrosomia (>4,000 g) among the racial/ethnic groups in the CNMI.

**Results:**

Indigenous CNMI mothers (Chamorros and Carolinians, hereinafter Chamorro/Carolinian) and other Pacific Islander mothers were significantly more likely to have a preterm birth than Chinese mothers (adjusted odds ratio [AOR] = 2.7; 95% confidence interval [CI], 2.0–3.6 for Chamorro/Carolinians and AOR = 2.9; 95% CI, 2.1–4.1 for other Pacific Islanders). Additionally, Chamorro/Carolinian mothers and other Pacific Islander mothers were also significantly more likely to deliver babies with macrosomia (AOR = 2.4; 95% CI, 1.7–3.5 and 2.3; 95% CI 1.4–3.6 respectively) than Filipino mothers.

**Conclusion:**

Although underlying causes for these disparities are still unknown, these findings add to the limited knowledge on maternal and neonatal health among Pacific Islanders and provide support for further research and intervention development to aid in reducing racial/ethnic disparities of perinatal health in the CNMI.

## Introduction

The Commonwealth of the Northern Mariana Islands (CNMI) is a US island territory in the northwestern region of the Pacific Ocean. The CNMI is a chain of 14 islands with almost all inhabitants residing on 3 of the islands: Saipan, Tinian, and Rota. These islands have a total population of approximately 54,000 and are home to diverse races/ethnicities, which include the indigenous CNMI Pacific Islanders (Chamorros and Carolinians), other Pacific Islanders (Palauans, Marshallese, Chuukese, Pohnpeians, Kosraeans, Yapese, Samoans, and Hawaiians), Asians (Filipinos, Chinese, Japanese, Koreans, Thais, Indians, Bangladeshi, and Nepalese), and other races and nationalities (American whites, African Americans, and Russians) (1). In addition to the resident population, about 300,000 to 400,000 tourists visit the islands each year (2). Approximately 1,000 births occur in the CNMI annually among CNMI residents and nonresident tourists combined (3).

The perinatal health status of Pacific Islander women and their newborns in the CNMI is understudied. Studies conducted in other island jurisdictions show that indigenous Pacific Islanders overall appear to have poorer health outcomes than non-Pacific Islander populations, including increased risk of chronic diseases such as cancer, diabetes, and obesity (4,5). Additionally, research among Pacific Islander immigrant communities in the United States and elsewhere shows that Pacific Islander populations have a higher incidence of adverse birth outcomes such as preterm birth (<37 weeks) (6), low birth weight (<2,500 g) (7), macrosomia (>4,000 g) (7), and infant mortality (8) than non-Pacific Islander groups. The health disparities among Pacific Islanders found across other locations suggest similar disparities may exist in the CNMI. A CNMI report on noncommunicable diseases found that indigenous groups (Chamorro/Carolinian) and other Pacific Islanders had a high prevalence of chronic conditions, including obesity, diabetes, and high cholesterol (9). Additionally, the report observed that CNMI Pacific Islanders were more likely to have unhealthy behaviors such as using tobacco, chewing betel nut, and drinking alcohol than non-Pacific Islander groups (9). Chronic disease and unhealthy behaviors are known risk factors associated with adverse perinatal outcomes, suggesting that Pacific Islanders in the CNMI may be at higher risk for adverse perinatal health outcomes than non-Pacific Islander groups in the CNMI.

To the best of our knowledge only one study has been published on the perinatal health of CNMI residents (10). That study reported that the incidence of stillbirths in the CNMI was highest among Carolinians and other Micronesians and 3 times higher than that of Asians. Although research on Pacific Islander maternal and infant health is limited, some studies have demonstrated that disparities exist and should be explored further, especially in locations such as the CNMI, where Pacific Islanders are the indigenous population.

In a preliminary CNMI Maternal and Child Health Program report, we examined data on CNMI maternal and infant health and observed evidence of racial/ethnic perinatal health disparities in the CNMI. According to this report, indigenous groups and other Pacific Islanders in the CNMI had a higher prevalence of preterm birth (CNMI indigenous groups, 8.9% and other Pacific Islanders, 9.8%) and macrosomia (CNMI indigenous groups, 4.4% and other Pacific Islanders, 4.4%) compared with other racial/ethnic groups inhabiting these islands (5.7% for preterm birth and 2.8% for macrosomia) (3). The purpose of this study was to explore the racial/ethnic disparities that exist among Pacific Islander women residing in the CNMI and their newborns to encourage further research into the causes of adverse perinatal outcomes in this population and to promote targeted interventions to reduce preterm birth, low birth weight, and macrosomia.

## Methods

We conducted a retrospective cohort study by using data from delivery logs that documented births from 2007 through 2014 at the Commonwealth Healthcare Corporation (CHCC), the only hospital in the CNMI located on the island of Saipan. These deliveries consisted of births among CNMI residents and women residing on neighboring islands, Tinian and Rota, and children born to nonresident tourists. We were able to capture delivery data on women from neighboring islands because they fly to Saipan close to time of delivery to give birth at CHCC.

Information entered into the delivery logs was collected by CHCC’s Labor and Delivery Unit staff after delivery and was based on patient self-report and information from the patient’s medical chart. Gestational age was determined by self-report of last menstrual period and was conﬁrmed by ultrasound. Data were available for 8,918 live births. We excluded births with missing data on key risk factors (maternal ethnicity, n = 74; maternal age, n = 27, and number of antenatal care visits, n = 155). Newborns whose records were missing data on gestational age (n = 124) and birth weight (n = 33) were also excluded. Lastly, plural births were excluded (n = 78), leaving 8,427 live singleton births in the ﬁnal data set.

Delivery was classiﬁed as preterm (<37 weeks) or full-term (≥37 weeks). An infant was categorized as low birth weight (<2,500 g), normal birth weight (2,500–4,000 g), or high birth weight (> 4,000 g). Women were categorized by their self-identified race/ethnicity (Chamorro/Carolinian, other Pacific Islander, Filipino, Chinese, or other non-Pacific Islander), maternal age at time of delivery (<20 y, 20 y to <35 y, or ≥35 y), and number of antenatal care visits during pregnancy (0 visits, 1–8 visits, or ≥9 visits). Determination of the sufficient number of antenatal care visits was based on the literature (11).

SAS version 9.4 (SAS Institute, Inc) was used for statistical analysis. We conducted χ^2 ^tests for all selected demographic and outcome categorical variables by race/ethnicity. Multivariate logistic regression was performed to determine predictors of the selected outcomes by using best-ﬁt practices. For preterm birth, the maternal race/ethnicity reference group was Chinese, and for macrosomia, the maternal race/ethnicity reference group was Filipino. We changed the reference group in the 2 regressions to better contrast the findings across groups.

Selection of variables for inclusion in models was based on the literature (12) and the availability of predictors provided by CHCC delivery logs. The multivariate models controlled for race/ethnicity, maternal age, and number of antenatal care visits during pregnancy. For models in which birth weight was the outcome of interest, we examined only full-term singleton infants. Other potentially important information on risk factors for adjustment, such as paternal factors or maternal smoking, were not available in our data. Because nonresident tourist births were included in analyses, sensitivity analysis was conducted to examine results after excluding non-CNMI resident births. We defined nonresident births as those births by tourists who were visiting the CNMI at the time of their delivery. Resident births were those by permanent CNMI residents and contract workers who enter the CNMI to work indefinitely and contribute to its economy. The Commonwealth Healthcare Corporation approved this project as an institutional review board proxy, because a formal board does not exist in the CNMI.

## Results

The largest racial/ethnic group of women giving birth in the CNMI was the indigenous group, Chamorro/Carolinian (33.2%); 26.2% of women self-identified as Filipino, 20.7% as Chinese, and 9.3% as other Pacific Islander (Table 1). The majority of births were to women aged 20 to 34 (70.9%) and to those who had 1 to 8 antenatal care visits during their pregnancy (56.2%). Teen pregnancy (<20 y) was more prevalent among Chamorros/Carolinians (17.2%) and other Pacific Islanders (11.6%) than among Filipinos (5.1%), Chinese (0.7%), and other non-Pacific Islanders (1.7%), (*P* < .001).

**Table 1:** Characteristics and Outcomes, by Race/Ethnicity, Among Births (N = 8,427)^a^ in the Commonwealth Nation of the Mariana Islands, 2007–2014

Characteristic	Total, No. (%)	Race/Ethnicity, No. (%)					
		Chamorro/ Carolinian	Other Pacific Islander	Filipino	Chinese	Other Non-Pacific Islander	*P *Value^b^
**Total**		2,799 (33.2)	785 (9.3)	2,207 (26.2)	1,742 (20.7)	894 (10.6)	NA
**Maternal age at delivery, y**							
<20	710 (8.4)	480 (17.2)	91 (11.6)	112 (5.1)	12 (0.7)	15 (1.7)	<.001
20–34	5,975 (70.9)	2,056 (73.4)	571 (72.7)	1,351 (61.2)	1,329 (76.3)	668 (74.7)	
≥35	1,742 (20.7)	263 (9.4)	123 (15.7)	744 (33.7)	401 (23.0)	211 (23.6)	
**Number of antenatal care visits**							
0	1,520 (18.0)	436 (15.6)	151 (19.2)	382 (17.3)	419 (24.0)	132 (14.8)	<.001
1 to 8	4,737 (56.2)	1,533 (54.8)	486 (61.9)	1,133 (51.3)	1,041 (59.8)	544 (60.8)	
≥9	2,170 (25.8)	830 (29.6)	148 (18.9)	692 (31.4)	282 (16.2)	218 (24.4)	
**Gestational age**							
Full-term (≥37 wk)	7,870 (93.4)	2,575 (92.0)	710 (90.4)	2,045 (92.7)	1,678 (96.3)	862 (96.4)	<.001
Preterm (<37 wk)	557 (6.6)	224 (8.0)	75 (9.6)	162 (7.3)	64 (3.7)	32 (3.6)	
**Birth weight at full-term^a^**							
Normal (2,500–4,000 g)	7,344 (93.3)	2,375 (92.2)	655 (92.2)	1,911 (93.5)	1,592 (94.9)	811 (94.1)	<.001
Low (<2,500 g)	232 (3.0)	76 (3.0)	21 (3.0)	86 (4.2)	19 (1.1)	30 (3.5)	
High (>4,000 g)	294 (3.7)	124 (4.8)	34 (4.8)	48 (2.3)	67 (4.0)	21 (2.4)	

a For analysis of birth weight, preterm births were excluded and only full-term singleton births were included; thus, the sample size for this analysis was 7,870.

b Determined by χ^2 ^test; significant at *P =* .05.

Among all singleton deliveries, 6.6% were preterm (<37 weeks) (Table 1). Among all full-term singleton deliveries, 3.0% of babies were low birth weight and 3.7% had macrosomia. Preterm births were more prevalent among Chamorro/Carolinian women (8.0%) and other Pacific Islander women (9.6%) than among Filipino women (7.3%), Chinese women (3.7%), and other non-Pacific Islander women (3.6%) (*P* < .001). Additionally, we found a significantly higher prevalence of babies with macrosomia among Chamorro/Carolinian women (4.8%) and other Pacific Islander women (4.8%) than among Filipinos (2.3%), Chinese (4.0%), and other non-Pacific Islanders (2.4%), (*P* < .001).

In adjusted multiple logistic regression models, the odds of a Chamorro/Carolinian woman having a preterm birth were 2.7 times greater than for a Chinese woman (*P* < .001), and the odds of an other Pacific Islander woman having a preterm birth were 2.9 times greater than for a Chinese woman (*P* < .001) (Table 2). Younger women (<20 y) and older women (≥35 y) also had significantly higher odds of preterm delivery (younger women, adjusted odds ratio [AOR] = 1.3, *P* = .048; older women, AOR = 1.5, *P* < .001) than women aged 20 to 34. Additionally, women with no antenatal care (0 visits) or insufficient antenatal care (1–8 visits) were significantly more likely to have a preterm birth (AOR = 3.9, *P* < .001 and AOR = 2.8, *P* < .001, respectively) than women with sufficient antenatal care (≥9 visits).

**Table 2:** Crude and Adjusted Odds Ratios for Preterm Delivery Among Singleton Births (N = 8,427) in the Commonwealth Nation of the Mariana Islands (CNMI), 2007–2014

Risk Factor^a^	Preterm Delivery Among All CNMI Births^b^			Preterm Delivery Among CNMI Resident Births^b^		
	Crude OR (95% CI)	Adjusted OR^b^ (95% CI)	*P *Value^c^	Crude OR (95% CI)	Adjusted OR^b^ (95% CI)	*P *Value^c^
**Maternal race/ethnicity**						
Chamorro/Carolinian	2.3 (1.7–3.0)	2.7 (2.0–3.6)	<.001	2.0 (1.4–2.9)	2.2 (1.5–3.2)	<.001
Other Pacific Islander	2.8 (2.0–3.9)	2.9 (2.1–4.1)	<.001	2.4 (1.6–3.7)	2.3 (1.5–3.6)	<.001
Filipino	2.1 (1.5–2.8)	2.3 (1.7–3.1)	<.001	1.8 (1.2–2.7)	1.9 (1.3–2.8)	.002
Chinese	1.0 [Reference]	1.0 [Reference]	NA	1.0 [Reference]	1.0 [Reference]	NA
Other non-Pacific Islander	1.0 (0.6–1.5)	1.1 (0.7–1.7)	.76	1.0 (0.6–1.8)	1.2 (0.7–2.0)	.59
**Maternal age at delivery, y**						
<20	1.6 (1.2–2.1)	1.3 (1.0–1.8)	.048	1.4 (1.1–1.9)	1.3 (1.0–1.7)	.07
20–34	1.0 [Reference]	1.0 [Reference]	NA	1.0 [Reference]	1.0 [Reference]	NA
≥35	1.4 (1.2–1.8)	1.5 (1.2–1.9)	<.001	1.4 (1.1–1.7)	1.4 (1.1–1.8)	.002
**Number of antenatal care visits**						
0	3.5 (2.6–4.8)	3.9 (2.9–5.3)	<.001	3.8 (2.8–5.1)	3.7 (2.7–5.1)	<.001
1 to 8	2.6 (2.0–3.4)	2.8 (2.1–3.7)	<.001	2.9 (2.2–3.8)	2.9 (2.2–3.8)	<.001
≥9	1.0 [Reference]	1.0 [Reference]	NA	1.0 [Reference]	1.0 [Reference]	NA

Abbreviations: CI, confidence interval; NA, not applicable; OR, odds ratio.

a The control group for the preterm birth model is term births (≥37 weeks).

b Each risk factor is adjusted for all other variables in the table.

c Values are significant at *P* = .05.

The odds of a Chamorro/Carolinian woman having a baby with macrosomia was 2.4 times greater than for a Filipino woman (*P* < .001) and the odds of an other Pacific Islander woman was 2.3 times greater than for a Filipino woman (*P* < .001) (Table 3).

**Table 3:** Crude and Adjusted Odds Ratios for Macrosomia Among Singleton Births (N = 7,890) in the Commonwealth Nation of the Mariana Islands (CNMI), 2007–2014

Risk Factor^a^	Macrosomia at Full-Term Among All CNMI Resident Births^b^			Macrosomia at Full-Term Among CNMI Resident Births^b^		
	Crude OR (95% CI)	Adjusted OR^b^ (95% CI)	*P *Value^c^	Crude OR (95% CI)	Adjusted OR^b^ (95% CI)	*P *Value^c^
**Maternal race/ethnicity**						
Chamorro/Carolinian	2.1 (1.5–2.9)	2.4 (1.7–3.5)	<.001	2.0 (1.4–2.8)	2.4 (1.7–3.4)	<.001
Other Pacific Islander	2.1 (1.3–3.2)	2.3 (1.4–3.6)	<.001	2.1 (1.3–3.2)	2.3 (1.4–3.6)	<.001
Filipino	1.0 [Reference]	1.0 [Reference]	NA	1.0 [Reference]	1.0 [Reference]	NA
Chinese	1.7 (1.2–2.4)	1.6 (1.1–2.4)	.01	2.1 (1.4–3.2)	2.1 (1.3–3.2)	.001
Other non-Pacific Islander	1.0 (0.6–1.7)	1.0 (0.6–1.7)	.90	1.0 (0.5–2.0)	1.1 (0.6–2.0)	.87
**Maternal age at delivery, y**						
<20	0.3 (0.2–0.7)	0.3 (0.1–0.6)	<.001	0.3 (0.2–0.7)	0.3 (0.2–0.6)	<.001
20–34	1.0 [Reference]	1.0 [Reference]	NA	1.0 [Reference]	1.0 [Reference]	NA
≥35	1.0 (0.8–1.4)	1.3 (0.9–1.7)	.12	1.1 (0.8–1.4)	1.3 (1.0–1.8)	.09
**Number of antenatal care visits**						
0	1.2 (0.9–1.7)	1.2 (0.8–1.7)	.38	1.5 (1.1–2.2)	1.4 (1.0–2.1)	.05
1–8	1.0 (0.8–1.4)	1.0 (0.8–1.4)	.84	1.1 (0.8–1.5)	1.1 (0.8–1.5)	.62
≥9	1.0 [Reference]	1.0 [Reference]	NA	1.0 [Reference]	1.0 [Reference]	NA

Abbreviations: CI, confidence interval; NA, not applicable; OR, odds ratio.

a The control group for the macrosomia model is birth weights from 2,500 g to 4,000 g.

b Each risk factor is adjusted for all other variables in the table.

c Values are significant at *P* = .05.

In sensitivity analyses that excluded non-CNMI resident births (Table 2), the odds of preterm birth remained elevated for Chamorro/Carolinian women (AOR = 2.2; 95% CI ,1.5–3.2) and other Pacific Islanders (AOR = 2.3; 95% CI, 1.5–3.6). For macrosomia, the exclusion of non-CNMI residents did not change results appreciably (<10% change in effect estimates) (Table 3).

## Discussion

To our knowledge, ours is the first study to examine racial/ethnic disparities associated with adverse perinatal outcomes among Pacific Islanders in this unique remote island territory. Understanding disparities in maternal and neonatal health in Pacific Island nations and territories is necessary to implement and target interventions for women and their infants residing in these locations. Little literature focusing on maternal and neonatal health of Pacific Islanders has been published, and even less literature exists on adverse perinatal outcomes among Pacific Islanders residing in small remote islands such as the CNMI, where Pacific Islander populations are indigenous. Our study showed that the odds for preterm birth and macrosomia were higher among Pacific Islanders residing in the CNMI than for non-Pacific Islander populations. These findings are consistent with studies of Pacific Islanders conducted in other locations (6,7).

The higher odds of preterm birth and macrosomia among Pacific Islander women in the CNMI may be due to underlying causes and lifestyle factors, such as maternal tobacco use, maternal betel nut use, pre-eclampsia, maternal stress, pre-pregnancy or maternal obesity, gestational diabetes, and lack of or poor antenatal care. Maternal tobacco or betel nut use during pregnancy are known to cause preterm birth (13,14). Analysis from the CNMI’s Non-Communicable Disease Hybrid Survey showed that Pacific Islander women of childbearing age use betel nut more (Chamorro, 32%; Carolinian, 65%; other Pacific Islander, 56%) than all other racial/ethnic groups (2%). In addition, this analysis showed that current smoking was highest among Chamorros (41%) (9). Although we did not know the incidence of smoking during pregnancy among the women in our study, international data suggest 20% to 50% of female smokers attempt to quit or reduce their smoking during pregnancy, with half of these women succeeding and the remainder cycling through attempts to cut down and quit followed by relapsing (15). These data suggest that these behaviors may play a role in the high prevalence of preterm births found among the CNMI’s Pacific Islander women. Studies from other regions also report that using tobacco and betel nut while pregnant is prevalent among indigenous and Pacific Islander women (16,17), further suggesting the possibility of their use as a reason for preterm births among Pacific Islanders.

Other factors including pre-eclampsia and stress have also been linked with increased risk for preterm birth (18,19). A study from Hawaii found that Pacific Islander women were at higher risk for pre-eclampsia than white women (20), suggesting that Pacific Islander women in the CNMI also may have higher incidence of pre-eclampsia. No data on these conditions in the CNMI exist, and we were unable to obtain such information. Stress, another potential cause for preterm birth, has been reported to be higher among indigenous and Pacific Islander women than among women of nonindigenous or non-Pacific Islander background (21).

Like the potential underlying causes of preterm birth, potential underlying causes of macrosomia in the CNMI may be pre-pregnancy obesity, maternal obesity, and gestational diabetes (22,23). Although we did not have information on whether participants in our study had these conditions, the CNMI’s Non-Communicable Disease Hybrid Survey found that Pacific Islanders had a much higher prevalence of overall overweight and obesity (9) (Chamorro/Carolinian, 83%; other Pacific Islanders, 76%) than all other racial/ethnic groups in the CNMI (Filipino, 55.0%; other Asian, 40.5%; other races/ethnicities, 62.2%), and also a higher prevalence of diabetes (Chamorro/Carolinian, 20%; other Pacific Islanders, 15%) than non-Pacific Islanders (Filipino, 8.7%; other Asian, 5.4%; other races/ethnicities, 8.6%) (9). These data suggest that high birth weights among Pacific Islanders may be linked to maternal obesity or gestational diabetes. In addition, other studies have demonstrated that the prevalence of gestational diabetes and obesity is higher among Pacific Islanders than among other racial/ethnic groups (24,25).

Insufficient antenatal care is another risk factor for preterm birth and macrosomia (26). Studies have shown that having a sufficient number of antenatal care visits, early initiation of these visits, and higher quality of prenatal care leads to a healthier pregnancy and reduces maternal, fetal, and neonatal morbidity (including preterm birth and unhealthy birth weight) and mortality (11,26). Lower levels of prenatal care in our study may have been due in part to the existence of only one maternity hospital in the CNMI, which is located on Saipan. The outer islands, Tinian and Rota, have small health clinics that do not employ midwives or obstetrician/gynecologists, resulting in delays in identifying and treating pregnancy complications. Hence, residents of the more remote Pacific islands have geographic barriers to health care that are similar to those reported among rural US residents (27). These limited resources can affect the frequency of antenatal care visits and increase the risk for adverse birth outcomes, more so than among indigenous Pacific Islanders on these remote islands than among nonindigenous groups. Although antenatal care among other Pacific Islanders was lower than among other racial/ethnic groups, their risk for preterm birth and macrosomia was still higher even after controlling for the number of antenatal care visits in our regression analysis. Further analyses are needed to better understand how fewer antenatal care visits may be associated with the risk for preterm birth and macrosomia among Pacific Islanders in the CNMI.

Socioeconomic factors may also may influence preterm birth and high birthweight among Chamorros, Carolinians, and other Pacific Islanders. Factors such as income and education attainment are associated with adverse perinatal outcomes (28). According to 2010 CNMI census data, Carolinians and other Pacific Islanders had a lower proportion of high school graduates or people with an equivalent education and a lower proportion of bachelor degrees than other CNMI racial/ethnic groups (1). In contrast, Chamorros had the second largest median income among these groups, and their educational attainment was about 20% higher than that of Carolinians and other Pacific Islanders in CNMI (1). The low socioeconomic status of Carolinians and other Pacific Islander groups could be a contributor to their higher risk of preterm birth and macrosomia; however, it does not seem to explain the higher risk among Chamorros. More research is needed to understand this.

A strength of this study is that there is only one hospital in the CNMI where all deliveries occur, hence our data should be considered population-based. We do not have information on the number of home births in the CNMI. Another strength is that this research is one of a limited number of studies that focus on perinatal health among Pacific Islanders, and it is the first to present findings on racial/ethnic disparities in adverse perinatal outcomes in the CNMI. In addition, studies and reports often group Pacific Islanders with Asians, masking important health outcomes within groups of more populous Asian ethnicities. It is important to stratify these populations, because large cultural and lifestyle differences exist between Asians and Pacific Islanders, which ultimately influence their health.

Our study had limitations. One was the limited availability of health data in the CNMI. Also, because of limited resources and low epidemiologic capacity in these islands, we found a limited number of covariates that could be included in our analysis. Data on more variables must be routinely collected and must be more accessible to adjust for more factors, such as sociodemographics, mother’s island residence (Saipan, Tinian, or Rota), maternal substance use (including tobacco and betel nut), maternal obesity, gestational diabetes, and pre-eclampsia. In addition to the negative perinatal outcomes evaluated, it would have been useful to explore other outcomes such as maternal and neonatal morbidity. However, these data are not consistently collected and recorded, and we would probably have been limited in our statistical power. Qualitative studies could also help shed light on these disparities. The CNMI has few health care resources and does not have the same health workforce capacity as the mainland United States. As a result, lack of health care access, non-use of health services, and low health knowledge among Pacific Islander women should also be studied to understand barriers that may cause increased preterm birth and macrosomia among Pacific Islander groups.

Prevalence of low birth weight in our study, at 3% across groups, was low compared with what is reported in other Pacific Island nations, where the range is 6% to 13% (29). This low rate is particularly notable given the high levels of tobacco and betel nut use among indigenous CNMI women of childbearing age. Birth weights differ across countries, and it is possible that indigenous Pacific Islander infants naturally have higher birth weights than infants in other regions. It has been suggested that 3,000 g should be considered the threshold for low birth weight among Pacific Islanders residing in Australia (30). If the higher birth weights seen in the CNMI were related to healthy childhood outcomes, there would be less concern for possible negative health effects. Yet the high prevalence of preterm birth we observed combined with elevated levels of diabetes and overweight reported in the CNMI (9) suggest these infants should be followed to determine if their birthweights are related to elevated risk of adverse childhood outcomes such as asthma, diabetes, and overweight.

Lastly, our results slightly changed after excluding nonresident tourist births in the sensitivity analysis. Visa restrictions in the CNMI are less strict than those in the mainland United States, which allows more citizens of other countries to fly to the CNMI to give birth, thereby making their infants US citizens. Tourist births accounted for 16.5% of all births in the CNMI during the study period, with 91% of those births among Chinese tourists, 8% among other Asian groups, and the remaining 1% among other races/ethnicities (3). Because of these tourist births, we excluded more than half of Chinese births in our sensitivity analysis, and the proportion of Filipino births, which tends to be among low-income mothers (1) with higher odds of preterm birth, increased. A literature search did not find any studies on the sociodemographics of children of Asian tourists born in the United States, but we hypothesize that this group probably includes wealthier mothers who presumably had access to high-quality prenatal care in their country of origin, before arrival in the CNMI. In an earlier study, we observed lower incidence of preterm birth (4.4%) among tourists compared with nontourists (8.0%) (3). Because nonresident tourists fly to the CNMI near the time of delivery, they would be less likely to deliver preterm. Our sensitivity analysis, which excluded nonresident births, indicated about a 20% decrease in the odds of preterm birth among Chamorro/Carolinians and Pacific Islanders.

The health consequences of preterm birth and macrosomia are well documented (12,17,26,29). This study was the first to examine racial/ethnic disparities in adverse perinatal outcomes in the CNMI. In light of our findings, we recommend that maternal child health interventions be tailored for the cultural needs of Pacific Islander groups in the CNMI. A more in-depth understanding of underlying factors driving these disparities would inform strategies for antenatal care access and would increase support for healthy pregnancies and family planning. By providing public health programs and clinical services with strategies to target indigenous women and other Pacific Islander women in the CNMI, we can work toward closing this disparity gap and increasing healthy perinatal outcomes for all women in the CNMI.
